# Effects of serum hypersensitive C-reactive protein and BMI on cognitive dysfunction in first-episode and drug-naive patients with major depressive disorder

**DOI:** 10.1186/s12888-026-07782-y

**Published:** 2026-01-27

**Authors:** Jingping Wu, Yuanyuan Huang, Hongxin Lu, Hehua Li, Sumiao Zhou, Zhendong Zhang, Ziyun Zhang, Shixuan Feng, Chenyu Liu, Lam Mei Fong, Kai Wu, Fengchun Wu

**Affiliations:** 1 Department of Psychiatry, The Affiliated Brain Hospital, Guangzhou Medical University, No. 36 Ming Xin Road, Liwan District, Guangzhou, Guangdong 510370 China; 2 Guangdong Engineering Technology Research Center for Translational Medicine of Mental Disorders, Guangzhou, Guangdong 510370 China; 3 Department of Psychiatry, Longyan Third Hospital of Fujian Province Department of Psychiatric Medicine, Longyan, Fujian 364030 China; 4 Psychiatric Service of the Centro Hospitalar Conde de São Januário, Macao, 999078 China; 5 School of Biomedical Sciences and Engineering, South China University of Technology, Guangzhou International Campus, Guangzhou, Guangdong 511442 China

**Keywords:** Major depressive disorder, Cognitive dysfunction, Serum hypersensitive C-reactive protein, Body mass index

## Abstract

**Background:**

Major Depressive Disorder (MDD) is among the most prevalent psychiatric disorders worldwide, contributing substantially to global disease burden and disability. Besides its core symptoms, the cognitive dysfunction in MDD patients seriously impairs their social functioning and warrants attention. Cognitive dysfunction in MDD may be related to demographic characteristics, serum inflammatory indices levels and Body Mass Index (BMI, calculated as weight divided by height squared, in kg/m²). This study focuses on the cognitive dysfunction and its associated factors in first-episode, drug-naive patients with MDD.

**Methods:**

The study enrolled overall 116 first-episode, drug-naive patients with MDD and 100 healthy controls (HC) for comparison. Demographic information was obtained from all participants. We used the Chinese version of the MATRICS Consensus Cognitive Battery (MCCB) to assess cognitive function and the 17-item Hamilton Depression Rating Scale (HAMD-17) to evaluate MDD symptoms. Levels of serum inflammatory indices, such as hypersensitive C-reactive protein (hs-CRP), leukocyte (WBC), neutrophil, and eosinophil were measured. Subsequently, multiple linear regression analysis was utilized to determine factors linked to cognitive dysfunction across the five domains with MDD patients.

**Results:**

In this study, MDD patients exhibited significantly poorer cognitive function across five domains - speed of processing (SOP), attention/vigilance (AV), working memory (WM), verbal learning (VIS), and visual learning (VRB) - compared with HC (*p* < 0.001). Their serum levels of hs-CRP (*p* = 0.022), WBC (*p* = 0.015), and neutrophil (*p* < 0.001) were significantly elevated than HC, whereas the level of eosinophil (*p* = 0.031) was significantly lower. The results of Spearman correlation analysis indicated that BMI was connected to cognitive function among MDD patients, specifically in the domains of SOP (*r* = 0.274, *p* = 0.003) and AV (*r* = 0.189, *p* = 0.042). Multiple linear regression analysis indicated that education years and hs-CRP level were significantly influenced by the cognitive function in the VIS domain among patients with MDD.

**Conclusion:**

Our study shows a potential link between serum hs-CRP levels, BMI and cognitive dysfunction in MDD patients. This finding establish serum hs-CRP and BMI as robust, clinically accessible correlates of cognitive dysfunction in MDD, supporting their potential utility as complementary indicators for identifying patients at highest risk of cognitive impairment.

**Clinical trial number:**

Not applicable.

## Introduction

Major Depressive Disorder (MDD) is characterized by significant and enduring low mood, which distinguishes it as a mental illness [1, 2]. It is typified by high prevalence, disability, recurrence, and suicide rates, inflicting considerable harm on patients’ physical and mental health [3]. Beyond core symptoms like low mood and diminished interest, cognitive dysfunction represents a crucial clinical feature of MDD [4]. Cognitive function, an advanced brain function covering learning, memory, thinking, attention, and decision-making [5]. Cognitive dysfunction is highly prevalent among patients with MDD, primarily affecting memory, attention, and executive function [6, 7]. Among these, impairments in attention and executive function, which are associated with frontal lobe dysfunction, are particularly prominent [8, 9]. Cognitive impairment in MDD is present throughout the entire disease course, persisting even during periods of remission and worsening with disease recurrence. This persistence and exacerbation of cognitive dysfunction increase the complexity of treatment [5, 7, 10–12]. In our preliminary research, we have identified that serum indicators such as homocysteine [13, 14] and antioxidants [5, 15] are closely associated to cognitive function. Many studies indicate educational level [16], serum inflammatory factors [17–19] and Body Mass Index (BMI, calculated as weight in kilograms divided by the square of height in meters, in kg/m²) are significant contributors affecting cognitive dysfunction in MDD patients. A positive correlation exists between educational level and cognitive function [20]. Lower-educated individuals with MDD are more likely to have impaired cognitive functions, such as impairment in language memory [16]. Inflammation and BMI also play crucial roles in cognitive dysfunction.

Research evidence suggests that inflammation may significantly contribute to cognitive dysfunction in MDD [21]. Activation of peripheral inflammation can affect the brain’s immune-inflammatory system via the blood brain barrier (BBB) [22]. Inflammation inhibits neurogenesis by reducing neuronal proliferation and cell survival, which is associated with structural and functional abnormalities in the hippocampus, as well as deficits in verbal ability and memory [23]. Alternatively, inflammation may affect cognitive function by decreasing striatal reward-related neural activation, which is linked to corticostriatal circuit dysfunction [24–27]. Chronic long-term inflammation, signified by elevated levels of pro-inflammatory cytokines, may induce neuroinflammation or neurodegeneration, thereby contributing to cognitive deficits [28, 29]. Serum inflammatory measures, which reflect peripheral inflammation, are closely associated with cognitive impairment in depression. Specifically, there is an inverse relationship between patients’ cognitive function and levels of Hypersensitive C-reactive protein (hs-CRP), particularly in terms of executive function and psychomotor speed [30]. Similarly, across other mental illnesses, cognitive deficits are closely associated with serum inflammatory indices levels. In schizophrenia (SCZ), for instance, levels of white blood cells (WBC) are associated with impairments in processing speed, language learning, and working memory [31]. In Alzheimer’s Disease (AD), patients with cognitive dysfunction exhibit higher levels of neutrophils compared to those without cognitive impairment [32]. Animal models of AD demonstrate that neutrophils are involved in BBB disruption, which adversely affects memory function [33]. This evidence underscores that an imbalance in serum inflammatory indices is closely related to cognitive impairment.

BMI, a metric widely utilized to evaluate the correlation between weight and height, also functions as a comprehensive tool for assessing fundamental aspects of physical health. The influence of BMI on cognitive function is complex and multifaceted. A possible association exists between elevated BMI, particularly in cases of obesity, and cognitive impairment. Obesity has been recognized as a predictor linked to cognitive dysfunction. Earlier research has proposed a bidirectional relationship between obesity and cognitive function. Obesity is considered a significant contributor to cognitive decline, particularly in areas such as executive function, intellectual capacity, psychomotor performance and speed, and visuospatial abilities. Conversely, cognitive decline may also increase the risk of obesity [34]. However, some studies have presented entirely different perspectives on the potential association between obesity and cognitive impairment [35]. Prior research has noted that in specific populations, BMI is positively correlated with cognitive performance [36], highlighting its significance in influencing cognitive function.

Several studies have identified peripheral inflammatory mediators, including interleukin (IL)-8, as partial mediators of the connection between BMI and cognitive function in patients with MDD [19]. In a three-year longitudinal study of adolescents with depression, high BMI was found to potentially impair executive function through interleukin-6 [37]. This suggests that inflammation levels and BMI in MDD patients may be key factors influencing cognitive function.

To sum up, the educational level, serum inflammatory factors and BMI are related to cognitive function in MDD. However, a lack of standardized, accessible, and affordable clinical indicators for cognitive dysfunction remains, as these indicators are influenced by factors such as the disease course and pharmacological treatments. Therefore, this study aims to analyze the associations among cognitive function and education years, serum hs-CRP and BMI in first-episode, drug-naive MDD patients. It seeks to identify correlations and impacts on cognitive impairment, deepening our understanding of MDD cognitive pathology and treatment, and establishing quantification standards to detect cognitive impairment early and provide clinical interventions.

## Method

### Study design and participants

A cross-sectional study was performed involving 116 first-episode drug-naive MDD patients from the outpatient department of the Affiliated Brain Hospital of Guangzhou Medical University. Before the study commenced, researchers provided the participants with comprehensive information about the study’s objectives and procedures. After ensuring that the participants fully understood the study details, written informed consent was obtained. This study was approved by the Ethics Committee of the Affiliated Brain Hospital of Guangzhou Medical University and was conducted in accordance with the latest version of the Declaration of Helsinki (2013). All participants completed clinical data collection, cognitive function assessment, and blood sample collection at the hospital. The clinical symptoms of MDD patients were evaluated and diagnosed by two trained professional psychiatrists, in accordance with the criteria of the fifth edition of the Diagnostic and Statistical Manual of Mental Disorders (DSM-5). Participants were included in the study based on the following criteria: (1) fulfillment of the DSM-5 diagnostic criteria for MDD, HAMD-17 ≥ 17; (2) being a first-episode patient whose disease duration did not exceed 2 years from the time of onset; (3) having no prior use of psychiatric medications or having used them irregularly for less than 2 weeks; (4) being aged between 18 and 45 years and of Han ethnicity; and (5) having no antibiotic treatment in the past 3 months. Exclusion criteria encompassed: (1) severe physical illness; (2) presence of other psychiatric conditions or brain organic dysfunction; (3) a history of alcohol or other psychoactive substance misuse; (4) a history of head injury involving loss of consciousness or significant sequelae; (5) pregnancy or breastfeeding; (6) history of electroconvulsive therapy; and (7) inability to participate in cognitive assessments. Additionally, 100 college students and volunteers from nearby universities and communities were enrolled as healthy controls (HC) [38, 39]. These controls were matched by gender and age, and none had received antibiotic treatment in the past 3 months. Each HC was independently assessed by two board-certified psychiatrists using DSM-5 criteria to confirm the absence of lifetime psychiatric illness in the participant and first-degree relatives.

### Clinical measurements

In this study, general information was collected from all the first-episode and drug-naive MDD and HC individuals. This information included basic demographic details such as age, education years, marital status, occupation, and address, as well as physical measurements like height and weight. The 17-item Hamilton Depression Rating Scale (HAMD-17) [40] was used to assess the clinical psychiatric symptoms of MDD. The evaluation of the current and past-week history of first-episode, drug-naive MDD patients was conducted by experienced psychiatrists who had undergone consistency training and calibration in the HAMD-17.

### Evaluation of cognitive function

Drawing on previous studies, our research utilized the MATRICS Consensus Cognitive Battery (MCCB) to assess cognitive function in both groups [41, 42]. The analysis focused on five cognitive domains: processing speed (SOP), attention/ vigilance (AV), working memory (WM), verbal learning (VRB), and visual learning (VIS) [5, 15]. This approach to evaluating cognitive function in patients with severe mental illnesses has indeed become a standard practice in clinical research [43]. Cognitive functions of all participants were analyzed across these domains to identify significant deficits in individuals with MDD compared to those in the HC group.

### Analysis of serum inflammatory measures

All participants were required to fast for at least 8 h. Venous blood samples (5 mL) were obtained from each participant within 48 h following the completion of clinical evaluations. The blood samples were transported to the Laboratory of Affiliated Brain Hospital of Guangzhou Medical University within 30 min for processing. A professional technician performed centrifugation for 10 min to separate the serum, which was then used to measure levels of hs-CRP, leukocyte (WBC), neutrophil, and eosinophil. Hospital-trained clinical-laboratory technicians processed all biochemical assays under complete blinding to participant identity. Serum hs-CRP concentrations were quantified with a kit supplied by Beijing Leadman Biochemistry Co., Ltd (Beijing, China); total WBC, neutrophil and eosinophil counts were measured with reagents kits supplied by Shenzhen Mindray Bio-Medical Electronics Co., Ltd (Shenzhen, China). All experimental procedures were performed strictly following the manufacturer’s instructions.

### Statistical analysis

Statistical analysis for this study was performed using IBM SPSS Statistics 22.0 (IBM Corp., Chicago, USA), with a two-tailed p-value of less than 0.05 considered statistically significant. Categorical variables (such as gender) between MDD and HC groups were compared using chi-square tests, while continuous variables (such as age and education years) were compared with independent-samples t tests. Clinical characteristics, laboratory results, and cognitive scores were subjected to normality testing. Normally distributed variables (Shapiro-Wilk test, *p* > 0.05) were described as mean ± standard deviation (SD) and were compared across groups using one-way ANOVA. Non-normally distributed variables (Shapiro-Wilk test, *p* < 0.05) were described as median and interquartile range (median [IQR]), with Mann-Whitney U tests used for comparisons. Spearman correlation analysis was employed to explore relationships among serum inflammatory measures, clinical data, and cognitive performance. Multiple regression analysis (backward elimination model) with MCCB scores as the dependent variable was conducted to investigated the link between MDD cognitive function and serum inflammatory indices levels.

## Result

### Demographic and clinical characteristics

This investigation involved 216 participants in total, of whom 116 were first-episode, drug-naive patients with MDD, and the remaining 100 participants were HC. The demographic and clinical characteristics of these two groups are presented in Table 1. In this study, the mean age of the MDD group was 23.06 years (SD = 4.49), whereas the mean age of the HC group was 22.24 years (SD = 2.64). In the MDD group, 60.34% were female and 39.66% were male. No statistically significant differences were found in gender (χ^2^ = 2.279, *p* = 0.095) and age (*t* = -1.663, *p* = 0.098) between the MDD and HC groups. However, the two groups exhibited statistically significant differences in education years (*U* = 3973.500, *p* < 0.001) and BMI (*U* = 4669.500, *p* = 0.014). Medians (IQR) for education were 15 (12, 16) years in the MDD group and 16 (14, 17) years in HC; corresponding BMI values were 21.69 (19.80, 24.60) kg/m^2^ and 21.16 (18.82, 23.82) kg/m^2^. The mean HAMD-17 score of the MDD patients was 23.68 (SD = 4.93), with a mean age of first onset at 22.23 years (SD = 4.95) and a median total disease duration of ten months.

**Table 1:** The demographic and clinical characteristics of the two groups of participants

	MDD (*n* = 116)	HC (*n* = 100)	χ^2^/t/U	*p*
Gender(M/F)	46/70	51/49	2.279	0.095
Age (y)	23.06 ± 4.49	22.24 ± 2.64	-1.663	0.098
Education years (y)	15.00 (12.00, 16.00)	16.00 (14.00, 17.00)	3973.500	< 0.001^*^
BMI (kg/m^2^)	21.69 (19.80, 24.60)	21.16 (18.82, 23.82)	4669.500	0.014^*^
Age of first onset(y)	22.23 ± 4.95	-	-	-
Total disease duration(m)	10.00 (3.00, 18.00)	-	-	-
HAMD score	23.68 ± 4.93	-	-	-

*, *p*<0.05. Abbreviations: MDD, Major Depressive Disorder; HC, Healthy Controls; BMI, Body Mass Index; HAMD, The 17-item Hamilton Depression Rating Scale

### Cognitive function of MDD patients and HC

The MDD and HC groups exhibited significant differences across the five cognitive domains. The MDD group exhibited lower cognitive scores compared with the HC group in SOP, AV, WM, VRB, and VIS. Even after adjusting for age, gender and education years, the two groups remained significantly separated across all five cognitive domains (SOP, AV, WM, VRB, VIS) (all *p* < 0.05) (Table 2). After entering age, gender, education and BMI as covariates, between-group differences remained significant across all five cognitive domains (all *p* < 0.05).

**Table 2:** Cognitive function of MDD patients and HC

	MDD (*n* = 116)	HC (*n* = 100)	F	*p*	Adjusted F*	Adjusted p*
SOP	32.68 ± 9.68	45.70 ± 9.91	95.094	< 0.001	94.766	< 0.001
AV	33.28 ± 9.24	42.16 ± 8.62	52.736	< 0.001	53.285	< 0.001
WM	39.17 ± 11.66	47.36 ± 10.70	28.56	< 0.001	28.925	< 0.001
VRB	31.57 ± 9.73	41.72 ± 7.80	68.825	< 0.001	70.836	< 0.001
VIS	39.03 ± 9.21	45.36 ± 7.08	31.329	< 0.001	32.551	< 0.001

*Adjusted values were calculated with age, gender, education years as covariates

Abbreviations: MDD, Major Depressive Disorder; HC, Healthy Controls; SOP, Speed of Processing; AV, Attention/Vigilance; WM, Working Memory; VRB, Verbal Learning; VIS, Visual Learning

### The differences in serum inflammatory measures levels between the MDD and HC groups

As shown in Table 3, the MDD and HC groups exhibited substantial differences with respect to the levels of several serum inflammatory measures, including WBC (*U* = 4684.000, *p* = 0.015), neutrophil (*U* = 4186.000, *p* < 0.001), eosinophil (*U* = 4814.500, *p* = 0.031), and hs-CRP (*U* = 4753.000, *p* = 0.022). Specifically, the MDD group exhibited elevated median counts of WBC (MDD 6.35 [IQR 5.60, 7.98] vs. HC 6.05 [IQR 5.23, 6.98]×10⁹/L), neutrophils (3.85 [IQR 3.20, 5.18] vs. 3.30 [IQR 2.70, 4.30] ×10⁹/L) and hs-CRP (1.10 [IQR 1.10, 1.98] vs. 1.00 [IQR 0.53, 1.30] mg/L), whereas the median eosinophil count was significantly lower in MDD (0.09 [IQR 0.05, 0.17] vs. 0.13 [IQR 0.08, 0.19]×10⁹/L).

**Table 3:** Comparison of serum inflammatory measures levels between the two groups

	MDD (*n* = 116)	HC (*n* = 100)	U	Z	*p*
WBC	6.35 (5.60,7.98)	6.05 (5.23,6.98)	4684.000	-2.438	0.015^*^
neutrophil	3.85 (3.20,5.18)	3.30 (2.70,4.30)	4186.000	-3.526	< 0.001^*^
eosinophil	0.09 (0.05,0.17)	0.13 (0.08,0.19)	4814.500	-2.154	0.031^*^
hs-CRP	1.10 (1.10,1.98)	1.00 (0.53,1.30)	4753.000	-2.290	0.022^*^

*, *p*<0.05. Abbreviations: MDD, Major Depressive Disorder; HC, Healthy Controls; WBC, Leukocyte/White Blood Cells; hs-CRP, Hypersensitive C-reactive Protein

### Correlation of cognitive function with serum inflammatory measures and BMI in MDD and HC group

**Fig. 1:**
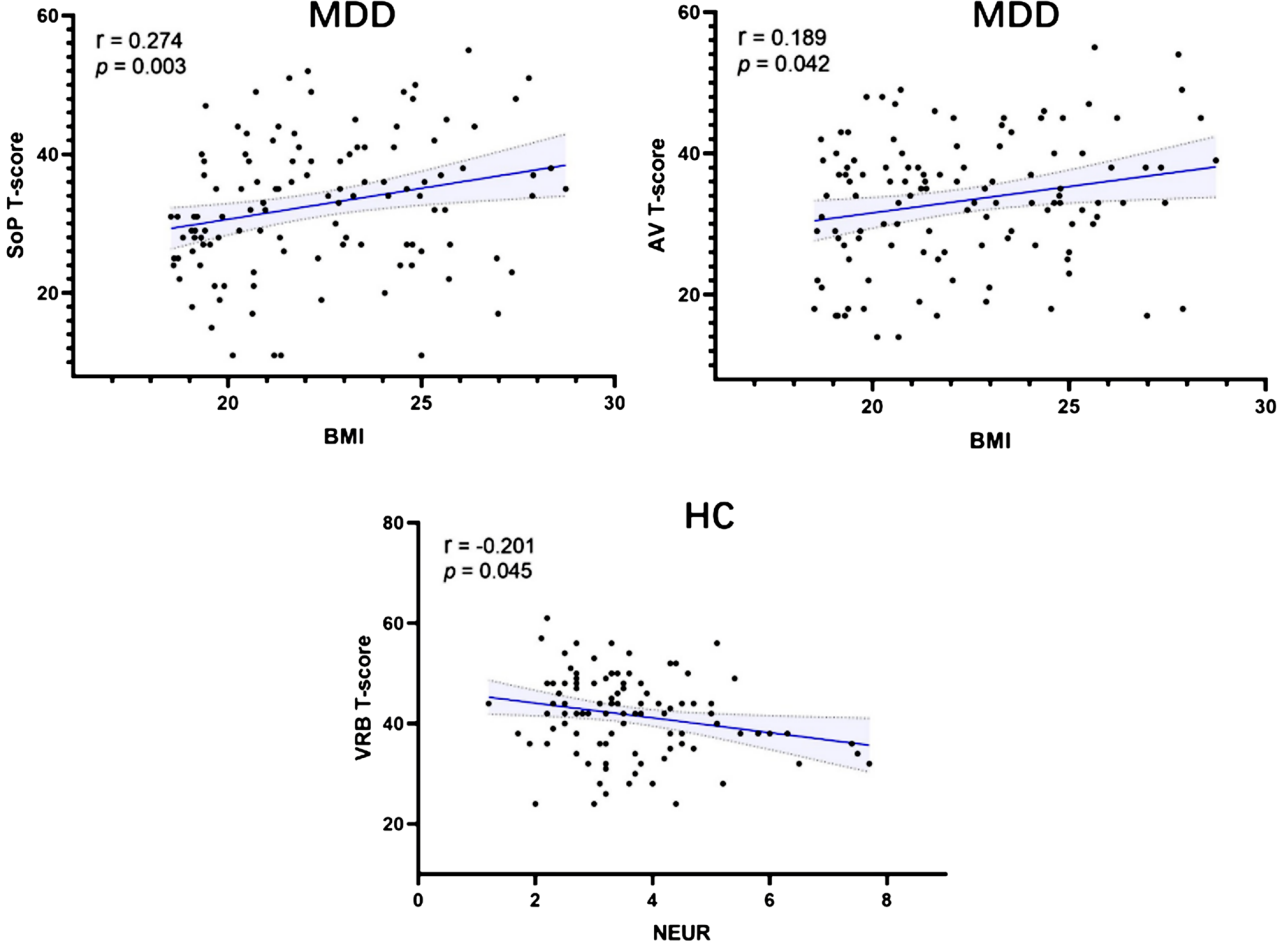
Analysis of correlations among cognitive domain T scores, BMI and serum inflammatory measures. The scatter plots, from left to right, depict the relationships between SOP and BMI in the MDD, AV and BMI in the MDD, and VRB, and neutrophil levels in the HC

After adjusting for age, gender and education years, significant between-group differences persisted across all five cognitive domains (all *p* < 0.05). Further analysis using Spearman correlation analysis revealed that in the HC, VRB was inversely correlated with neutrophil levels (*r* = -0.201, *p* = 0.045), but this did not survive multiple-comparison correction. In the MDD group, SOP showed a positive association with BMI (*r* = 0.274, *p* = 0.003), and AV also showed a positive association with BMI (*r* = 0.189, *p* = 0.042). However, no significant correlations were observed between other serum inflammatory indices and cognitive performance (*p* > 0.05). After multiple-comparison correction, only the SOP–BMI positive correlation survived (Table 4) (Fig. 1).

**Table 4:** Correlation between five dimensions of MCCB and serum inflammatory measures, as well as BMI in HC and MDD groups

			SOP	AV	WM	VRB	VIS
HC	BMI	*r*	0.034	-0.027	0.191	-0.004	-0.019
		*p*	0.739	0.790	0.057	0.972	0.849
		Adjusted *p*	1.000	1.000	0.285	1.000	1.000
	WBC	*r*	-0.013	-0.007	0.077	-0.164	-0.070
		*p*	0.894	0.945	0.447	0.103	0.490
		Adjusted *p*	1.000	1.000	1.000	0.515	1.000
	neutrophil	*r*	-0.015	0.038	0.071	-0.201	0.020
		*p*	0.882	0.707	0.483	0.045^*^	0.843
		Adjusted *p*	1.000	1.000	1.000	0.225	1.000
	eosinophil	*r*	-0.075	-0.077	-0.141	0.001	-0.112
		*p*	0.459	0.445	0.162	0.993	0.267
		Adjusted *p*	1.000	1.000	0.810	1.000	1.000
	hs-CRP	*r*	0.071	0.092	0.039	0.001	0.044
		*p*	0.483	0.365	0.704	0.995	0.661
		Adjusted *p*	1.000	1.000	1.000	1.000	1.000
MDD	BMI	*r*	0.274	0.189	0.097	0.169	0.140
		*p*	0.003^*^	0.042^*^	0.299	0.070	0.135
		Adjusted *p*	0.015^*^	0.210	1.000	0.35	0.675
	WBC	*r*	0.024	0.017	0.094	-0.030	0.005
		*p*	0.797	0.858	0.315	0.747	0.959
		Adjusted *p*	1.000	1.000	1.000	1.000	1.000
	neutrophil	*r*	0.033	-0.044	-0.009	-0.057	-0.031
		*p*	0.728	0.639	0.922	0.542	0.740
		Adjusted *p*	1.000	1.000	1.000	1.000	1.000
	eosinophil	*r*	0.100	0.065	0.078	0.016	-0.118
		*p*	0.283	0.486	0.406	0.862	0.205
		Adjusted *p*	1.000	1.000	1.000	1.000	1.000
	hs-CRP	*r*	0.076	-0.007	0.044	-0.023	-0.002
		*p*	0.416	0.943	0.636	0.810	0.980
		Adjusted *p*	1.000	1.000	1.000	1.000	1.000

*, *p*<0.05. Abbreviations: MDD, Major Depressive Disorder; HC, Healthy Controls; SOP, Speed of Processing; AV, Attention/Vigilance; WM, Working Memory; VRB, Verbal Learning; VIS, Visual Learning; BMI: Body Mass Index; WBC, Leukocyte/White Blood Cells; hs-CRP, Hypersensitive C-reactive Protein

### Regression analysis of serum inflammatory measures levels, BMI and cognitive function

This study employed multiple regression analysis with a backward elimination model to examine the influence of serum inflammatory indices and BMI on cognitive function in MDD patients. In our analysis, MCCB scores were used as the dependent variable, while demographic data, clinical features and serum inflammatory measures were included as independent variables. The results indicated that the regression model was significant for education years (B = 1.272, *t* = 3.758, *p* < 0.001) and hs-CRP levels (B = -1.654, *t* = -2.085, *p* = 0.039) in relation to VIS cognitive function performance (Table 5). However, no significant associations were observed between hs-CRP and the other four cognitive domains.

**Table 5:** Factors affecting cognitive function in patients with MDD

		B	S.E	t	*p*-value	95% CI	
						Lower	Upper
VIS	(constant)	14.756	8.870	1.664	0.099	-2.827	32.339
	Education years	1.272	0.338	3.758	< 0.001^*^	0.601	1.943
	hs-CRP	-1.654	0.793	-2.085	0.039^*^	-3.227	-0.081
	Gender	2.371	1.675	1.416	0.160	-0.949	5.691
	Age	-1.157	0.749	-1.545	0.125	-2.642	0.328
	Age of first onset	0.742	0.676	1.098	0.275	-0.598	2.083
	BMI	0.452	0.330	1.372	0.173	-0.201	1.106
	WBC	1.572	1.346	1.168	0.245	-1.097	4.242
	neutrophil	-1.354	1.495	-0.906	0.367	-4.318	1.610

*, *p*<0.05. Abbreviations: MDD, Major Depressive Disorder; VIS, Visual Learning; BMI: Body Mass Index; hs-CRP, Hypersensitive C-reactive Protein; WBC, Leukocyte/White Blood Cells

## Discussion

The primary results of this study are presented as follows: (1) The T-scores for the five cognitive domains were substantially reduced in the MDD group compared with the HC group. (2) Versus the HC group, the MDD group exhibited markedly elevated levels of WBC, neutrophil, and hs-CRP, as well as significantly reduced levels of eosinophil among the serum inflammatory measures. (3) Elevated serum hs-CRP levels were found to be strongly associated with cognitive impairment among individuals with MDD. (4) The MDD group had a markedly elevated BMI compared to the HC group. Additionally, BMI was positively correlated with cognitive function in MDD patients, particularly within the domains of SOP. (5) In the HC group, serum neutrophil levels exhibited a negative correlation with VRB, but this did not survive multiple-comparison correction. Currently, there is no definitive conclusion regarding the impact of serum inflammatory indices on cognitive function in MDD patients. Therefore, our study results will further elucidate the factors contributing to cognitive impairment in these patients and deepen our understanding of how serum inflammatory indices relate to cognitive decline in MDD.

Our study revealed that, contrasted with HC, MDD patients had significantly lower scores across all five dimensions of cognitive function. Age, gender and education-adjusted analyses still revealed significant between-group differences across all five cognitive domains, in line with our earlier findings [5]. Cognitive dysfunction in MDD can compromise social functioning, attention, learning capacity, and exacerbate depressive symptoms, all of which may influence treatment efficacy and patient prognosis. Cognitive dysfunction in MDD persists even during remission and worsens with each recurrence. Our findings further confirm multi-dimensional cognitive impairment in first-episode, drug-naive MDD patients. Cognitive function serves as an essential clinical feature of MDD and plays a crucial role in predicting and assessing future treatment outcomes.

Additionally, significant differences in serum hs-CRP, WBC, neutrophil, and eosinophil levels were found between the two groups. Compared to CRP, hs-CRP is more sensitive and can accurately detect low-concentration CRP levels. It is a trace protein in the blood, synthesized in large quantities by hepatocytes during infection or inflammation [44]. Depression is often associated with a pro-inflammatory phenotype, particularly in the central nervous system, where inflammatory conditions can lead to chronic neuroinflammation through the activation of microglia and astrocytes [45]. A review on the potential mechanisms underlying cognitive impairment highlighted that microglial activation can alter the expression of various neurotoxic mediators, promote the accumulation of inflammatory factors, and amplify inflammatory cycles, leading to glial damage and neuronal cell death [46].

The association between serum inflammatory indices and cognitive dysfunction has garnered increasing attention. In particular, serum hs-CRP levels have been highlighted as a significant factor in MDD-related cognitive impairment. Previous research has established a connection between elevated hs-CRP levels and cognitive dysfunction across various conditions, including MDD [30], mild cognitive impairment (MCI) [47], and SCZ [48]. These effects not only disrupt the hypothalamic energy homeostasis and appetite regulation centers but also impact other brain regions, including the prefrontal cortex and hippocampus, contributing to corresponding cognitive impairment [49]. This represents a critical factor contributing to progressive neuronal damage. Both acute and chronic systemic inflammation can lead to cognitive dysfunction. On one hand, hs-CRP triggers systemic inflammation, increasing proinflammatory cytokines and causing cognitive dysfunction. On the other hand, inflammation affects vascular reactivity. High hs-CRP levels cause cerebral vasodilation and reduced vascular reactivity, accelerating cognitive decline, particularly in executive function and daily living activities [46, 50]. Previous research has indicated that elevated levels of hs-CRP are linked to an increased likelihood of cognitive impairment [51]. In a longitudinal study spanning 12 years, higher serum hs-CRP levels were found to predict memory decline, suggesting hs-CRP as a simple and objective index for identifying cognitive dysfunction [52]. To explore the relationship between serum inflammatory levels and cognitive function in first-episode, drug-naive patients with MDD, we conducted a correlation analysis between serum inflammatory measures and cognitive domains. Nevertheless, no statistically significant association was observed between hs-CRP and cognitive function. Previous studies have shown that measuring epigenetic levels of CRP (DNAm CRP) in peripheral blood yields more stable inflammation levels and stronger correlations with cognitive impairment than serum CRP [53]. Therefore, the instability of hs-CRP in serum, which is susceptible to rapid concentration changes in plasma, may contribute to our results. Future research could consider measuring DNA methylation levels of more stable inflammatory measures to quantify their association with cognitive function. To further explore the relationship between serum inflammatory indices and cognitive function in first-episode, drug-naive patients with MDD, we conducted multiple linear regression analysis. Our findings reveal a potential link between elevated levels of hs-CRP and cognitive impairment in MDD patients, indicating that hs-CRP might act as a risk factor for cognitive deficits, particularly in the domain of VIS. The result indicates that systemic inflammation from high hs-CRP levels in MDD patients can cause cognitive dysfunction, similar to past reports on inflammation-induced cognitive dysfunction in other diseases [47, 54].

In addition to the findings regarding hs-CRP and cognitive function, our study demonstrated that MDD patients had a significantly higher BMI compared to HC, suggesting that patients with MDD are more likely to experience an increase in BMI. This observation aligns with previous research indicating a higher prevalence of obesity in MDD patients [18, 55]. However, relative to published sex-specific BMI norms for Chinese adults [56], our MDD group exhibited markedly lower values, corroborating the hypothesis that illness-associated appetite suppression drives weight loss in depression. The parallel reduction observed in our HC group plausibly reflects the contemporary emphasis on leanness among young people rather than a pathological process. To disentangle these influences we will launch a population-representative, longitudinal survey that prospectively enrols a larger first-episode, medication-naïve sample and models how age, education and graded BMI shape symptomatic trajectory and cognitive outcome over time. In further correlation analysis, we identified a positive association between BMI and cognitive dysfunction in MDD patients, particularly in the domain of SOP. This observation contrasts with many previous studies that identified obesity as a possible predictor for cognitive dysfunction [57–60]. We hypothesize that first-episode, drug-naive MDD patients might demonstrate reduced BMI levels, which could be attributed to anorexia and weight loss-related nutritional deficiencies. These deficiencies might also contribute to cognitive impairment in these patients. Biologically, the peripheral immune system and central inflammatory mediators communicate bidirectionally. An elevated BMI is associated with increased peripheral and central inflammation, a condition that can trigger the stimulation of brain immune cells, including microglia and astrocytes. Sustained immune-inflammatory activation can alter emotions and cognitive function [61]. Nonetheless, some studies offer different perspectives. For instance, a prior study has identified a significant positive association between BMI and cognitive performance in male patients with SCZ [36]. Additionally, some research suggested that a healthy BMI doesn’t adversely affect cognitive function [62]. In other neurodegenerative diseases with cognitive impairment, a reduction in leptin due to weight loss may lead to cognitive dysfunction [63]. Moreover, well-metabolized obesity in older patients may confer a certain protective effect against the pathological mechanisms of AD [64], which also supports our results to some extent. Prior work demonstrates that elevated BMI strata among patients with MDD predicted more pronounced cognitive impairment, indicating that adiposity stratifies cognitive performance within this population [19]. Moreover, sex moderates these deficits: males typically outperform females on spatial tasks, whereas females exhibit superior verbal performance, underscoring the need to consider both BMI and sex when characterizing cognition in MDD [65]. Importantly, because our research was restricted to young adults experiencing their first major depressive episode, the observed associations between BMI, inflammatory status and cognition cannot be extrapolated to individuals whose first episode occurs after mid-life. This age-bias is an intrinsic limitation of the present sample. In our forthcoming study we will enroll a substantially larger, age-stratified sample to delineate how age at onset influences cognitive performance and to dissect the respective effects of BMI and systemic inflammation on cognition across distinct onset-age groups.

Our results indicated a nominal negative correlation between neutrophil and VRB within the HC group that did not survive multiple-comparison correction. This association was absent in the MDD group, indicating that neutrophil is not a strong correlate of VRB in MDD. Previous research in non-demented cohorts has demonstrated that elevated neutrophil levels were linked with cognitive dysfunction and accelerated deterioration of episodic memory [66]. These findings collectively highlight the importance of maintaining peripheral immune homeostasis in protecting cognitive function. Future research could investigate this correlation in more diverse populations, both cross-sectionally and longitudinally, to better identify cognitive dysfunction risk factors [67, 68].

## Limitations

We acknowledge several limitations in our study. First, it is a cross-sectional analysis of MDD patients. Serum inflammatory measures fluctuate over time, and their long-term, stable impact on MDD patients’ cognitive function remains unclear. Second, although our study found that serum hs-CRP affects MDD patients’ cognitive function, the specific mechanism of action of serum inflammatory measures on cognitive function is still unknown. Therefore, in the future, we plan to expand the cross-sectional study of MDD patients and conduct follow-up assessments after pharmacological intervention. We will implement robust, reproducible assays for serum inflammatory measures and integrate rigorous experimental designs with multi-omics analyses to identify risk factors underlying cognitive dysfunction in MDD. Our ultimate goal is to identify robust inflammatory indicators that are closely associated with cognitive impairment in MDD patients and can be readily applied in clinical practice. Third, our sample was predominantly composed of young individuals, which may limit the generalizability of the findings to the broader depressive population. Future studies with expanded age ranges or a specific focus on particular demographics (e.g., college students) are warranted to further delineate the risk factors for cognitive impairment in MDD. Fourth, because participants were recruited across geographically distinct regions, residual confounding arising from region-specific lifestyles and dietary patterns may bias cognitive estimates. Future studies should prospectively quantify these variables and incorporate them into statistical models to clarify their independent contributions to depressive symptom severity and cognitive impairment in MDD.

## Conclusion

Our research reveals that MDD patients suffer from extensive cognitive dysfunction and exhibit markedly higher serum hs-CRP levels than HC. The observed association between elevated serum hs-CRP levels and reduced VIS performance in first-episode, drug-naïve MDD patients positions hs-CRP as a readily accessible inflammatory indicator of concurrent cognitive impairment in this population. Although BMI correlated with select cognitive domains in MDD, its value as an indicator of cognitive dysfunction requires confirmation in larger, longitudinal studies. In summary, our results show a close relationship between serum inflammatory indices, BMI and cognitive dysfunction in first-episode and drug-naive MDD patients, offering new directions for future research.

## Data Availability

The datasets generated during the current study are not publicly available, but can be requested from the corresponding author on reasonable request.

## Abbreviations

MDD: Major Depressive Disorder
HC: Healthy Controls
MCCB: The MATRICS Consensus Cognitive Battery
HAMD-17: The 17-item Hamilton Depression Rating Scale
hs-CRP: Hypersensitive C-reactive Protein
WBC: Leukocyte/White Blood Cells
SOP: Speed of Processing
AV: Attention/Vigilance
WM: Working Memory
VIS: Verbal Learning
VRB: Visual Learning
BMI: Body Mass Index
BBB: Blood Brain Barrier
SCZ: Schizophrenia
AD: Alzheimer’s Disease
DSM-5: The fifth edition of the Diagnostic and Statistical Manual of Mental Disorders
